# Incorporation of carbon black into a sonogel matrix: improving antifouling properties of a conducting polymer ceramic nanocomposite

**DOI:** 10.1007/s00604-023-05740-z

**Published:** 2023-04-04

**Authors:** Alfonso Sierra-Padilla, David López-Iglesias, Paloma Calatayud-Macías, Juan José García-Guzmán, José María Palacios-Santander, Laura Cubillana-Aguilera

**Affiliations:** 1 Department of Analytical Chemistry, Institute of Research on Electron Microscopy and Materials (IMEYMAT), Faculty of Sciences, Campus de Excelencia Internacional del Mar (CEIMAR), University of Cadiz, Campus Universitario de Puerto Real, Polígono del Río San Pedro S/N, 11510 Puerto Real, Cadiz, Spain; 2 Instituto de Investigación e Innovación Biomédica de Cadiz (INiBICA), Hospital Universitario ‘Puerta del Mar’, Universidad de Cadiz, 11009 Cadiz, Spain

**Keywords:** Conducting polymers, Nanomaterials, Sonogel-Carbon electrochemical sensors; Differential pulse voltammetry, Chlorophenols, Antifouling properties, High-energy ultrasound

## Abstract

A new electrochemical sensor device has been developed through the modification of a polyaniline-silicon oxide network with carbon black (CB). Enhanced electrical conductivity and antifouling properties have been achieved due to the integration of this cheap nanomaterial into the bulk of the sensor. The structure of the developed material was characterized using Fourier transform infrared spectroscopy, energy-dispersive X-ray spectroscopy, and scanning electron microscopy techniques. Cyclic voltammetry was used to characterize electrochemically the Sonogel-Carbon/Carbon Black-PANI (SNG-C/CB-PANI) sensor device. In addition, differential pulse voltammetry was employed to evaluate the analytical response of the sensor towards sundry chlorophenols, common environmental hazards in aqueous ecosystems. The modified sensor material showed excellent antifouling properties, which led to a better electroanalytical performance than the one displayed with the bare sensor. Notably, a sensitivity of 5.48 × 10^3^ μA mM^−1^ cm^−2^ and a limit of detection of 0.83 μM were obtained in the determination of 4-chloro-3-methylphenol (PCMC) at a working potential of 0.78 V (vs. 3 M Ag/AgCl/KCl), along with proficient values of reproducibility and repeatability (relative standard deviation < 3%). Finally, the analysis of PCMC was carried out in multiple validated water samples using the synthesized SNG-C/CB-PANI sensor device, obtaining excellent results of recovery values (97–104%). The synergetic effect of polyaniline and carbon black leads to novel antifouling and electrocatalytic effects that improve the applicability of this sensor in sample analysis versus complex conventional devices.

**Graphical abstract:**

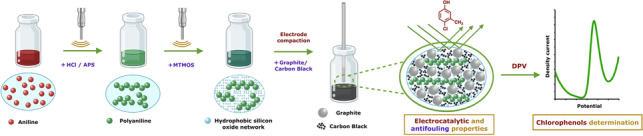

**Supplementary Information:**

The online version contains supplementary material available at 10.1007/s00604-023-05740-z.

## Introduction

The development of electrochemical sensor devices is currently devoted to simpler, cheaper, and more eco-friendly routes of synthesis of novel materials with enhanced analytical performance. Sonogel-Carbon electrodes (SNG-C), characterized by the application of high-energy ultrasound for sonocatalyzing the sol-gel process, stand out among handmade carbon ceramic sensors. Not only does this method of synthesis provide a highly competitive sensor through an eco-friendly and fast procedure, but a material susceptible to be modified with organic-inorganic receptors and biological recognition species to enhance its electroanalytical performance as well [1]. Whereas this modification of the naked SNG-C material can be carried out after the preparation of the sensor, by depositing the modifier in the electrode surface, it is possible to merge the modifiers with the silicon oxide network during its synthesis to obtain a completely novel bulk material. Hence, the compounds susceptible to be used as modifiers range from metal nanoparticles [2] or conducting polymers [3] to massive modifiers, providing new sensor devices with analytical applications in the sensing of diverse analytes and great results in pharmaceutical [4] or environmental [3] samples.

Notably, conducting polymers and carbon nano allotropes are two promising types of compounds employed to improve conductivity, electrocatalytic effect, and/or antifouling properties in sensor devices [5–7]. On the one hand, conducting polymers have been extensively applied recently in several devices such as electronic skins [8] or gas [9], among many others. Polyaniline (PANI) stands out due to its high electrical conductivity, good stability, and low cost, specifically in emeraldine salt form [10]. The constitution of thickness-controlled PANI coatings over supported electrodes is widely used for electrochemical sensing purposes. Despite their great applicability, degradation and fouling phenomena upon their use are reported in the literature, decreasing their lifetime [11].

On the other hand, carbon nanoallotropes have arisen in the last decades due to their properties and versatility, such as high-conductivity carbon black (CB). Remarkably, its employment as massive modifier of ceramic sensors has been exhaustively reported in recent years. CB stands out due to its electrocatalytic effect and low cost, among other interesting qualities [12, 13]. Usually, CB is used as filler of sensor transductor composites. For instance, the preparation of carbon paste electrodes (CPE) with CB has been previously reported, using either solid or liquid paraffin. They showed better electroanalytical performance than graphite electrodes in the determination of several analytes, such as nitrates or ascorbic acid [14, 15]. Moreover, CB-based CPEs have been used as platforms for tyrosinase biosensors with excellent performances towards catechol or phenol [15, 16], and for a gold nanoparticle-modified laccase biosensor used for the determination of hydroquinone in complex samples [17]. Besides, the manufacturing of novel nanocomposites and (bio) sensor devices with emerging technologies has arisen as an alternative in the last years. An interesting overview of the literature of CB-based electrochemical sensors devices has been issued recently by Arduini et al. [18]. Some interesting paper-based devices with CB have been tested in the analysis of capsaicin in pepper samples [19] or for the detection of hydrogen peroxide in aerogel phase, an interesting approach for low-cost monitoring of vaporized disinfectants [20]. Alternatively, novel press-printed CB films have been proposed as potential tools in microfluidics and other related micro- and nanochemistry applications [21], such as the amperometric determination of phenyl carbamate pesticides in environmental samples [22]. Finally, it is remarkable the use of CB in the fabrication of nanocomposites on paper substrates and its application as solid-state supercapacitors for energy storage [23].

Recently, a new electrochemical sensor device, namely Sonogel-Carbon-Polyaniline (SNG-C-PANI), has been developed through the modification of the silicon oxide network of sonogel with PANI synthesized by sol-gel route assisted by high-energy ultrasound. The direct modification of the bulk material allows avoiding the typical problems of sensors modified with polymer coatings, such as the fast degradation of the layer and the adsorption of large molecules derived from the redox process. Moreover, this sensor has been successfully applied in the determination of chlorophenols; however, some disadvantages can be noticed, such as fouling phenomena after performing successive analysis and high capacitance values [3]. Chlorophenols are listed in the priority pollutant lists of the European Union and US Environmental Protection Agency (USEPA) due to their high ecotoxicity and potential carcinogenicity, as well as their low biodegradation rate [24].

In this work, the massive modification of the original SNG-C-PANI bulk material with CB has been carried out, namely as Sonogel-Carbon/Carbon Black-Polyaniline (SNG-C/CB-PANI). CB has been selected as a modifier due to its low cost-effectiveness (c.a. 1 €/Kg), interesting electroanalytical properties, and absence of treatment requirement. The CB was successfully dispersed through the silicon oxide network. An improvement in the electrochemical performance of the sensor was expected with this modification thanks to its enhanced electrical conductivity and anti-fouling properties. The changes in the electroactive surface produced by fouling phenomena can affect electron-transfer kinetics and electrocatalysis, diminishing consequently the analytical sensitivity, stability, and operational lifetime [25]. No reports are found in the bibliography about the minimization of fouling phenomena in sensors employed to determine chlorophenols.

The structural and electrochemical characterization of the resulting SNG-C/CB-PANI material was carried out as well. The developed devices were tested with different chlorophenols, mainly 4-chloro-3-methylphenol or p-chloro-m-cresol (PCMC). The repeatability of the electrochemical measurements was studied with the modified sensor and compared with the unmodified one (with no CB). Finally, the developed sensor was successfully applied for the determination of PCMC in multiple untreated water samples, validating the results with HPLC as a reference technique. It must be highlighted the novelty of this work relies on that the proposed sensor device is simple, fast, eco-friendly, and cost-effective from the fabrication point of view. Moreover, other interesting features, such as versatility, stability, and easy mechanical renovation of the electrode surface also contribute to the performance of this new nanocomposite-based sensor. Finally, electrocatalytic and antifouling properties due to synergetic effect between PANI and CB in the composite could be considered as the main original contribution of this piece or research to improve electrical conductivity and electroanalytical performance, as well as to develop reliable and direct sample analysis. This kind of devices could be suitable to the analysis of several chlorophenols pollutants without significant interferences.

## Materials and methods

### Reagents and materials

All reagents employed were of analytical grade and used as received without further purification. Methyltrimethoxysilane (MTMOS), 4-chloro-3-methylphenol (PCMC), 4-chlorophenol, 2,4-dichlorophenol, and ascorbic acid were purchased from Merck (Darmstadt, Germany). Ammonium persulfate (APS), potassium hexacyanoferrate (II), phenol, catechol, hydroquinone, resorcinol, and bisphenol A were from Sigma Aldrich (Sigma, Steinheim, Germany). Aniline and 2,4,6-trichlorophenol were from Honeywell (Charlotte, NC, USA). Graphite powder was from Alfa Aesar (Johnson Matthey GmbH, Germany) and carbon black powder (CB) (*d* = 19–25 nm) from Cabot (Boston, MA, USA). All solutions were prepared with nanopure water obtained by passing twice-distilled water through a Milli-Q system (18 ΜΩ cm, Millipore, Bedford, MA). Glass capillary tubes, i.d. 1.15 ± 0.05 mm, were used as the bodies of the electrodes.

### Instrumentation

The synthesis of PANI and sonogel materials were carried out using a high-energy ultrasound generator, Sonicator 4000 MISONIX (MISONIX, Inc. Farmingdale, NY, USA) equipped with a 13-mm-diameter titanium tip, which provides 600 W as maximum output power. Electrochemical studies were made using an Autolab PGSTAT 20 potentiostat/galvanostat (Ecochemie, Utrecht, the Netherlands) connected with a personal computer and a 663 Metrohm VA Stand module. Processing data was made using GPES (General Purpose Electrochemical System) software. The measurements were carried out in a three-electrode electrochemical cell at room temperature, with the following composition: Ag/AgCl/KCl 3 M as reference electrode, platinum wire as counter electrode, and the Sonogel-Carbon-PANI and Sonogel-Carbon/CarbonBlack-PANI electrodes (geometric area: 1.04 × 10^−2^ cm^2^) as the working electrodes.

Fourier-transform infrared spectra (FTIR) were recorded with a Fourier-transform infrared spectrophotometer IRAffinity-1S (Shimadzu, Kyoto, Japan). X-ray diffractogram (XRD) were recorded with a diffractometer D8 Advance A25 (Bruker, Billerica, MA, USA) using as source Cu Kα λ = 0.1542 nm and operating with 40 kV and 30 mA. Samples were prepared appropriately for DRX analysis. On the one hand, the sonosols of electrode materials prepared according to the procedure given in the “Preparation of SNG-C/CB-PANI electrode material” section were placed in a cylindrical holder and dried at room temperature for one day. On the other hand, PANI dispersion was centrifuged for 5 min at 4000 rpm and the sediment was dried in an oven for 24 h at 100 °C.

Scanning electron microscopy (SEM) was carried out on a Nova NANOSEM 50 (FEI Company, Hillsboro, OR, USA), operating at 30 kV. For the SEM characterization, the filled tip of glass capillary tubes of SNG-C-PANI and SNG-C/CB-PANI electrodes were cut and inserted in the SEM sample holders with a hole previously drilled on its surface.

### Preparation of SNG-C/CB-PANI electrode material

The synthesis of SNG-C/CB-PANI was performed following a fabrication route previously reported with some modifications [3]. Firstly, to prepare the PANI dispersion, a precursor mixture containing 18 μL of aniline and 982 μL of 0.25 M APS and 1 M HCl solution was sonicated for 60 s at 40% of the ultrasound amplitude. Secondly, 120 μL of the synthesized dispersion was mixed with 480 μL of MTMOS and sonicated for 40 s at the same amplitude. Afterwards, 500 mg of a mixture of graphite and CB powder were added to the sonosol and homogeneously dispersed.

To prepare the CB/graphite mixture, 1 g of a mixture of both materials was homogenized in a vortex mixer for 30 s at 3000 rpm. In this regard, several percentages of CB, ranging from 2.5 to 15% were assessed.

The fabrication of the working electrodes was made following this methodology. Capillary tubes were filled with the mixture after the addition of graphite powder. Afterwards, carbon material was compacted and left to dry for 1 day. Before using, the electrode surface was polished gently using a Struers P1200 silicon carbide paper (Germany). Finally, a copper wire was inserted inside the capillary tubes to establish the electrical contact.

### Electrochemical measurements

For the measuring procedure, a three-electrode electrochemical cell with 25 mL of solution was used. A previous characterization was carried out via cyclic voltammetry (CV) in presence of 5 mM potassium hexacyanoferrate (II) in 0.5 M potassium nitrate to select the best electrode material configuration in terms of electrochemical performance. As instrumental parameters, 50 mV·s^−1^ of scan rate and potential range from –0.2 to +0.6 V were set up. The electroactivity of the incorporated PANI was evaluated by means of CV in 1 M HCl at 50 mV·s^−1^ and a potential window ranging between –0.2 and +1.2 V.

Differential pulse voltammetry (DPV) was chosen as the electroanalytical technique for the electrochemical determination of PCMC. The instrumental parameters were as follows: 8 mV as step potential, 50 mV of modulation amplitude, 0.4 s as interval time, and 0.05 s of modulation time. The current peaks obtained at 0.78 V (vs. Ag/AgCl/KCl 3 M) of working potential were evaluated considering a linear tangent baseline. To begin with, three buffer solution media at different pH were used to determine the optimal conditions for the detection of the target chlorophenol. The optimization study of optimal pH can be found in section S1 of Electronic Supplementary Material. Besides, in section S2 of supplementary information, capacities study can be found as well.

Thereafter, calibration plots in presence of different PCMC concentrations ranging from 2 to 10 μM were recorded by adding adequate volumes from 1 mM PCMC stock solution, using DPV with the above-mentioned parameters. After adding the corresponding aliquot to the cell, the resulting solution was stirred for 30 s. Subsequently, the corresponding differential pulse voltammogram was recorded. The measurements for each concentration were done in triplicate to estimate the repeatability of the sensor. Likewise, this procedure was used in the calibration with other chlorophenols.

Additionally, a study of the influence of different inorganic salts and electroactive species on the electrochemical signal of 7 μM PCMC oxidation was carried out. Particularly, 700 μM concentration of interferent was used to investigate the effect of sodium and calcium chloride, potassium and magnesium nitrate, and calcium and sodium carbonate on the analytical response of the device, whereas 70 μM concentration of cadmium and mercury chloride was also employed. A concentration of 7 μM was used for phenol, catechol, resorcinol, hydroquinone, ascorbic acid, and bisphenol A.

### Spiked water sample analysis

Several water samples were collected from different sources: tap water, wells of Chiclana de la Frontera and El Puerto de Santa María, Igualeja’s spring, and Los Hurones reservoir (all of them in Andalusia, Spain). The analysis of PCMC-fortified water samples was carried out as follows: water sample was spiked with 25 μM of the analyte. Afterwards, the sample was diluted 10 times with ABS solution at pH 4 to reach 2.5 μM. Subsequently, electrochemical analysis with DPV was carried out employing a standard addition method by using concentrations ranging from 1 to 5 μM. Lastly, the electrochemical measurements were carried out in triplicate.

Alternatively, the concentration of each sample was determined by HPLC and taken as reference values for comparison purposes. The used methodology can be found in section S3 from Electronic Supplementary Material.

## Results

### Preliminary studies for the optimization of SNG-C/CB-PANI materials

Firstly, as an initial attempt to modify the bare material (SNG-C-PANI), 600 mg of graphite and CB mixtures with different percentages were added to the sonosol. A pulverulent consistency could be appreciated in all modified materials, even in formulations with low proportions of CB. This effect may be attributed to the high specific surface area of the CB nanomaterial. Hence, aiming to accomplish the mechanical and compactness requirements for the final electrode material, several formulations of SNG-C/CB-PANI with 500 mg of graphite/CB mixture with different percentages of CB ranging from 1 and 50% were prepared. During the fabrication process, it was observed that SNG-C/CB-PANI formulations with a proportion of CB higher than 10% displayed worse consistencies; thus, proportions of CB ranging from 2.5 to 10% were electrochemically tested. The evaluation of their electrochemical performance, as well as of the SNG-C-PANI material (reference), was carried out using hexacyanoferrate (II) ion as the electrochemical probe.

**Fig. 1:**
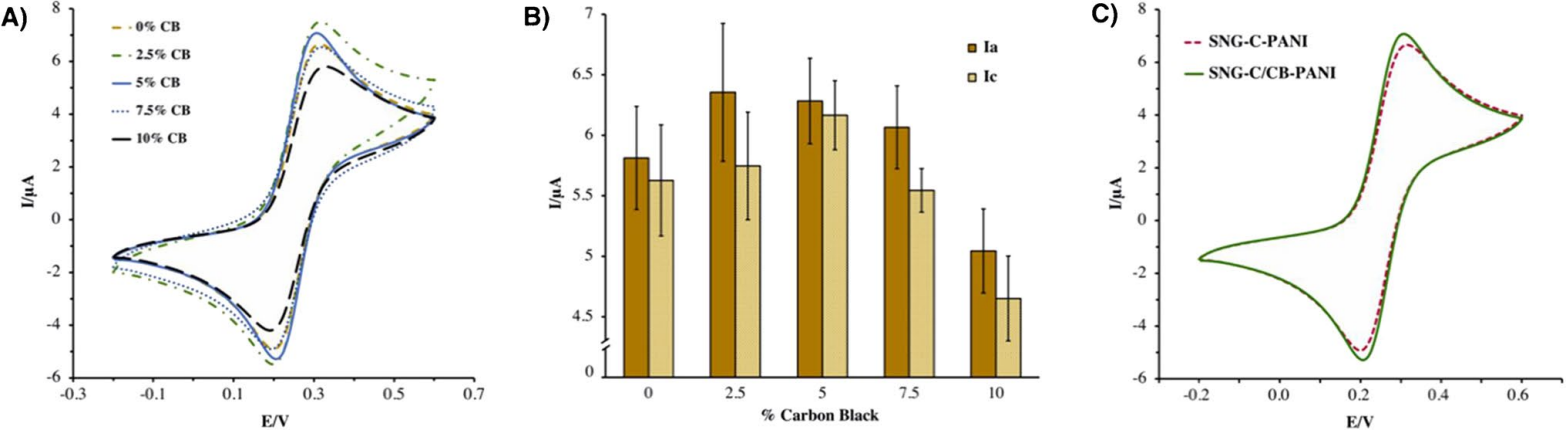
**A** Cyclic voltammograms and **B** bar chart of anodic and cathodic current peaks recorded with several electrode materials ranging their CB percentage in presence of 5 mM potassium hexacyanoferrate (II) in 0.5 M KNO_3_ at 50 mV·s^−1^. **C** Cyclic voltammograms recorded with two electrode materials, SNG-C-PANI and SNG-C/CB-PANI, in the same conditions

Two well-defined peaks corresponding to the oxidation and reduction processes of potassium hexacyanoferrate (II) were observed in the corresponding voltammograms (Fig. 1A). Particularly, the oxidation peak was found at 0.32 V, whereas the reduction peak was found at 0.20 V. Figure 1B shows intensity peak values obtained with each configuration. The highest anodic and cathodic intensity peak was achieved for the electrode with 5% of CB. The increase of the current peak with the CB percentage could be explained considering the higher conducting role of CB, as well as its nanostructured morphology. However, the highest current peak was reached at 2.5%. Based on this, it could be assumed that the electroactive area is decreased at higher proportions of CB, probably ascribed to a lower roughness area. The polyaniline/CB formulation with 5% CB showed higher reversibility as well, in terms of anodic and cathodic peak ratio, which indicates better electrochemical behaviour. Furthermore, low coefficient of variation can be also noticed with this formulation and hence, better reproducibility can be established. Therefore, the conducting material modified with 5% CB seems to be the best option as electrochemical sensor.

Additionally, Fig. 1C shows the cyclic voltammograms recorded with the best formulation modified with CB and the unmodified material in presence of 5 mM of potassium hexacyanoferrate (II). Interestingly, the modified material displays slightly better electrochemical response towards the benchmark analyte, in terms of anodic and cathodic current peaks, in comparison with the material without CB. In conclusion, the modification of the SNG-C-PANI material with 5% of CB led to a new nanocomposite with enhanced electrochemical features, able to be used as electrochemical transducer.

### Structural characterization

#### FTIR characterization

FTIR was used to examine the materials developed and corroborate the presence of PANI within the silicon oxide matrix. Hence, the infrared spectrum of powders from PANI (1), SNG-C (2), SNG-C/CB (3), SNG-C-PANI (4), and SNG-C/CB-PANI (5) materials was recorded (see Figure S1, Electronic Supplementary Material). Besides, assignments of the FTIR spectra bands are exposed in Table 1, as well as their comparison with the assignments of bands found in the literature [26, 27]. Importantly, the characteristic bands of PANI, C–N and C–C stretching in benzenoid and quinoid units of emeraldine, can be ascribed. In addition, spectra of carbon ceramic materials are remarkably similar, as expected, appearing bands of Si–CH_3_ symmetric deformation vibration at ≈ 1265 cm^−1^ (α), along with Si–O and Si–C stretching bands at ≈ 1110–1020 cm^−1^ (β) and ≈ 765 cm^−1^ (γ), respectively. These values are in consonance with the previous ones reported in the literature about Sonogel-Carbon materials [27]. Nonetheless, a higher intensity in the bands of Si vibration and stretching in the samples containing CB (samples 3 and 5) can be found. Interestingly, this may be attributed to the higher percentage of silane in the material [27]. Furthermore, the spectrum of PANI-modified materials (samples 4 and 5) exposes a low-intensity band at ≈ 1580 cm^−1^ and ≈ 1470 cm^−1^ that can be ascribed to C–N, C–C, and C=C stretching in the polymer [26]. Consequently, this fact demonstrates that PANI polymer has been successfully incorporated within the ceramic network in the material structure, regardless of the presence of CB.

**Table 1:** FTIR spectra assignments of the materials assessed

Sample	Band number/cm^−1^	Reference band number/cm^−1^ [26, 27]	Assignments
PANI	(a) 1580	1559	N=Q=N, C-N, C=C stretching in quinoid units
	(b) 1470	1481	π (C=C), C–C stretching in benzenoid units
	(c) 1295	1297	C–N stretching in sequential benzenoid and quinoid units
	(d) 1240	1240	C–N stretching in benzenoid units
	(e) 800	800	C–H deformation vibration
SNG	(α) 1265	1275	Si–CH_3_, symmetric CH_3_ deformation vibration
	(β) 1110–1020	1094–1029	Si–O–Si, Si–O stretching
	(γ) 765	771	Si–CH_3_, Si–C stretching

*PANI* polyaniline, *SNG* sonogel, *Q* quinoid

#### XRD characterization

Alternatively, diffractograms of PANI, SNG-C-PANI ,and SNG-C/CB-PANI materials were recorded using the X-ray diffraction technique, as can be noted in Figure S2 (Electronic Supplementary Material). Regarding the results registered, the peak intensities found in the diffractogram corresponding to PANI material are extremely low, in contrast with the sharp peaks evidenced in the diffractograms of the carbon ceramic composites. Indeed, the diffractogram of PANI is in consonance with the pattern of the orthorhombic polycrystalline structure of the polymer, according to the Joint Committee on Powder Diffraction Standards (JCPDS) card n° 53-1890 and 53-1891 databases. On the other hand, the peaks of modified and bare materials match with the pattern of hexagonal graphite registered in JCPDS card n° 75-1621. Furthermore, peaks found in PANI diffractogram were not distinguished in the ceramic composite diffractograms. Lastly, the material modified with carbon black displays the same crystallinity that the bare material, suggesting as expected that the amorphous carbon nanoallotrope does not change significantly the structure of the composite.

#### SEM characterization

Scanning electron microscopy (SEM) was used to study the electrode surface. For this purpose, micrographs of used and non-used SNG-C-PANI and Sonogel-C/CB-PANI electrodes were recorded and compared (Figure S3 of Electronic Supplementary Material). Interestingly, the separation between the electrode material and the capillary tube was almost negligible in non-used electrodes (Figures S3A and S3C), revealing a low volume contraction during the drying step. Nonetheless, this separation increased in the case of used SNG-C-PANI (Figure S3B) due to the erosion phenomena caused by electrochemical measurements. Similar observations on SNG-C electrodes have been reported previously [27]. Likewise, used SNG-C/CB-PANI (Figure S3D) also underwent this contraction but to a lower degree, which may be ascribed to a higher compactness due to the presence of CB [28].

Moreover, the surface of non-used electrodes (Figures S3E and S3G) presented a low number of holes and fissures. Notably, new holes and fissures were detected after several electrochemical measurements, whereas the spotted particles have been removed by the erosion. Remarkably, the amount of damage that appears in used electrodes (Figures S3F and S3H) is similar in both materials SNG-C-PANI and SNG-C/CB-PANI, suggesting that the effect of erosion is similar in both cases.

### Electrochemical characterization

**Fig. 2:**
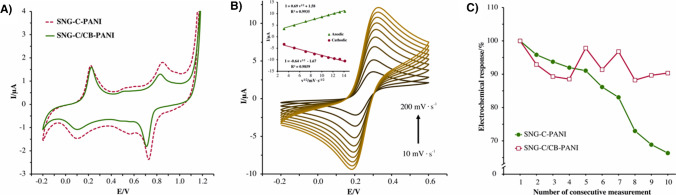
**A** Cyclic voltammograms recorded with a SNG-C-PANI (dashed line) and a SNG-C/CB-PANI materials (solid line) in 1 M HCl solution at 50 mV·s^−1^. **B** Cyclic voltammograms recorded with a SNG-C/CB-PANI electrode in presence of 5 mM potassium hexacyanoferrate (II) in 0.5 M KNO_3_ at the scan rates of 10, 25, 50, 75, 100, 125, 150, 175, and 200 mV·s^−1^. The inset shows the plot of anodic and cathodic peak current versus the square root of the scan rate. **C** Electrochemical responses of successive measurements recorded in presence of 7 μM PCMC with a Sonogel-Carbon-PANI electrode (•) and a Sonogel-Carbon Black/Carbon-PANI electrode (□) in ABS at pH 4. 0.5 M of KCl was used as supporting electrolyte

Firstly, SNG-C/CB-PANI and unmodified SNG-C-PANI materials were electrochemically characterized via CV. In both scenarios, the resulting cyclic voltammograms recorded in 1 M HCl solution (Fig. 2A) displayed four well-defined peaks, corresponding to the redox pairs of leucoemeraldine/emeraldine and emeraldine/pernigraniline. Furthermore, other non-defined peaks can be observed halfway between the main peaks, corresponding to intercrossed reactions. The peaks of the leucoemeraldine/emeraldine pair appear at similar potential with both materials, at 220 mV and 100 mV for oxidation and reduction processes, respectively. However, the peaks of the emeraldine/pernigraniline pair are located at lower potential values for the modified material, suggesting that CB may display an electrocatalytic effect in the redox reaction of polyaniline. Thus, the electroactivity of polyaniline in the modified material can be stated.

Finally, the electrochemical evaluation of SNG-C/CB-PANI material using hexacyanoferrate process as electrochemical probe was carried out accordingly to the conditions mentioned in the Experimental section 2.4. The results of these studies are displayed in Fig. 2B. As it can be appreciated, anodic and cathodic peaks were located at 0.32 V and 0.20 V, respectively, ascribed to the redox process of hexacyanoferrate (II) ion. The parameters of current and potential peaks observed at each scan rate are collected in Table S1. The anodic/cathodic peak current ratio is around 1 for all scan rate values. Furthermore, the peak potential did not increase with the scan rate. Both parameters can be ascribed to a high reversible system. Regarding the mechanism of the redox reaction, a linear relationship between both current peaks and the square root of scan rate (*R*^2^ = 0.994) suggests a diffusion-controlled process. Additionally, the value calculated for the slope of the plot of the logarithm of the anodic current peak versus the logarithm of the scan rate is 0.362 log A/log V·s^−1^, within 0.2 and 0.6 values, which corroborates a diffusion-controlled mechanism [29].

### Electrochemical performance of SNG-C/CB-PANI: electrochemical studies with 4-chloro-3-methyphenol (PCMC)

#### Study of fouling phenomena effect

The analytical performance of the SNG-C/CB-PANI material was assessed via DPV by using PCMC as the benchmark analyte. The response of the bare material, SNG-C-PANI, towards this analyte has been also investigated for comparison purposes. Figure 2C shows the electrochemical response of both materials after successive measurements. Remarkably, the peak current achieved with the new carbon black modified sensor remained practically unaltered after ten measurements, whereas the current peak decreases significantly in the case of SNG-C-PANI. In this regard, RSD values for SNG-C-PANI and SNG-C/CB-PANI are 13.9% and 4.6%, respectively. These results suggested that antifouling effect towards PCMC can be ascribed to the presence of CB in the modified material. The antifouling phenomena allow several electrochemical assays in a row without any further electrochemical and/or mechanical surface treatment.

#### Analytical calibration of different chlorophenols

**Fig. 3:**
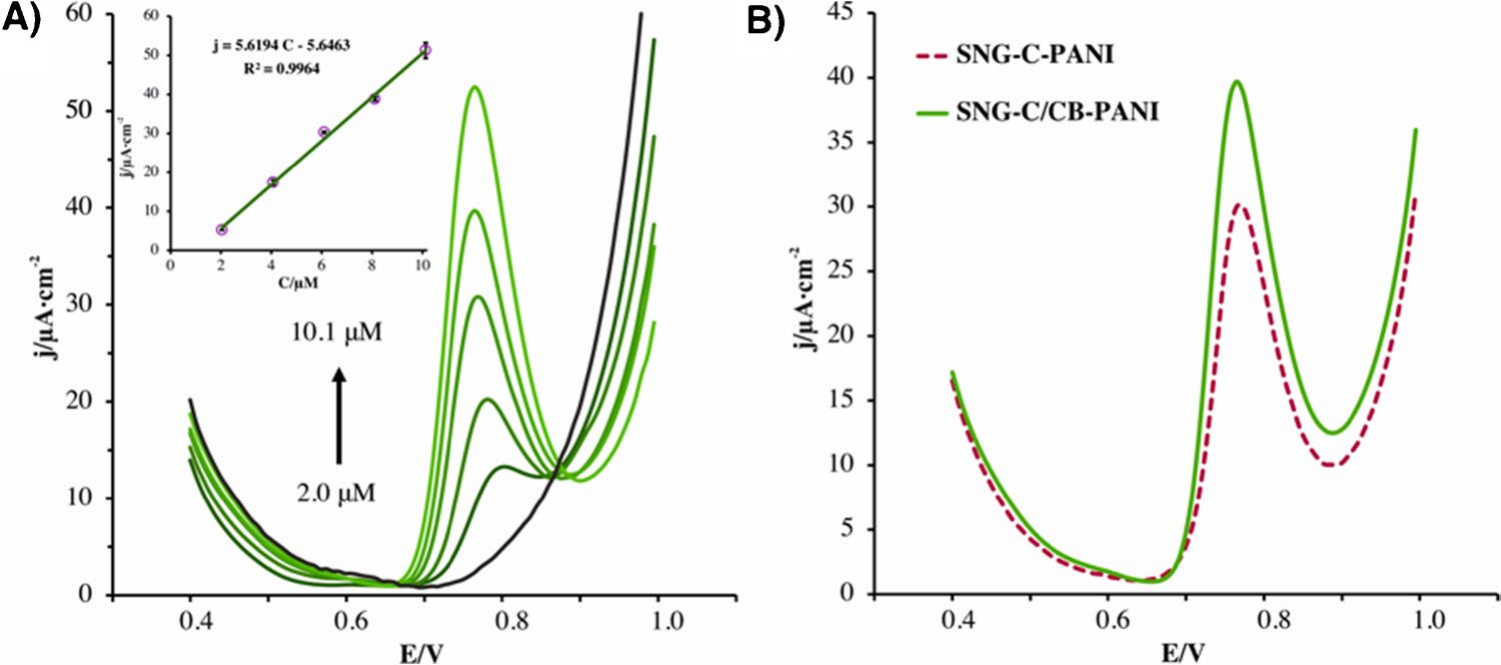
**A** Differential pulse voltammograms recorded with a SNG-C/CB-PANI electrode in presence of different concentrations of PCMC: 2.0; 4.1; 6.1; 8.1, and 10.1 μM. The inset displays the calibration plot obtained. **B** Differential pulse voltammograms recorded with a SNG-C-PANI (dashed line) and a SNG-C/CB-PANI (solid line) in presence of 8.1 μM of PCMC. The buffer solution was 0.1 M ABS at pH 4 with 0.5 M KCl. Modulation amplitude of 50 mV, step potential of 8 mV, and interval time of 0.4 s were the instrumental parameters

Analytical calibration of both the original and the modified materials was carried out using PCMC as the benchmark analyte. DPV was used as the electrochemical technique, employing the instrumental parameters mentioned in the experimental section 2.4. The calibration plot was represented by the density current anodic peak versus different concentrations of PCMC. Accordingly, multiple additions were made to reach concentrations ranging from 2 to 10 μM, measuring each concentration in triplicate. The oxidation peak of PCMC, at a working potential of 0.78 V (vs. Ag/AgCl/KCl 3 M), increased with each addition, as can be observed in Fig. 3. In addition, as can be noticed in the inset of this figure, a great linear relationship was established, with an excellent correlation coefficient (*R*^2^) of 0.996. Moreover, the sensitivity was calculated as the slope of the calibration curve. Therefore, for the SNG-C-PANI sensor the sensitivity was 3.05 × 10^3^ ± 3.03 × 10^2^ μA mM^−1^ cm^−2^, whereas for the modified SNG-C/CB-PANI sensor the sensitivity was 5.48 × 10^3^ ± 3.05 × 10^2^ μA mM^−1^ cm^−2^. The limit of detection (LOD) was also calculated as three times the standard deviation of the blank divided by the slope [30]. Hence, the LOD for Sonogel-Carbon-PANI was 1.79 ± 0.28 μM, whereas the modified sensor exhibits a LOD of 0.83 ± 0.13 μM. These values are comparable and one order of magnitude lower that the legal limits establish by the US Environment Protection Agency (USEPA), which specify a water quality criterion for PCMC as a concentration level of 500 μg/L (3.5 μM) [31]. Thus, both materials can be used to determine this analyte in quantities lower than the hazardous limits with great sensitivity.

It should be stressed that the developed SNG-C/CB-PANI sensor exhibits better quality analytical parameters towards PCMC than the unmodified SNG-C-PANI sensor. Thus, the presence of CB in the bulk material has meaningfully improved the electrochemical performance of the sensor, which can be attributed to an increase of the charge transfer. Furthermore, the low variabilities of the current density values between measurements obtained with the modified sensor can be ascribed to the minimization of the surface fouling, which is in consonance with what was observed in the “Study of fouling phenomena effect” section. Hence, the presence of CB avoids the surface fouling of the modified material suffered by other sensor devices. The improvement that emerges from the modification of the material reduces the analysis time as well, due to the no necessity of electrochemical or mechanical renewal of the sensor surface after each measurement and boosts the easiness of the determination procedure.

Besides, it is noteworthy to mention that the analytical parameters of the developed sensor are comparable to the results achieved with other sensors found in the literature. As shown in Table 2, outstanding sensitivity is accomplished with the modified material developed in this work, in comparison with the rest of the sensors reported. Moreover, the limit of detection is similar than those obtained with the rest of electrochemical sensors and, as discussed previously, it is competent with respect to the legal limits. Hence, additional steps of preconcentration or electrochemical renewal post-measurement are not required in the analysis of PCMC with the developed device, in contrast with the procedures reported in the literature. Thus, this difference implies lower time of analysis, simplicity, and the reduction of additional error sources. Moreover, the developed material can be produced using a cheap, easy, and green-friendly route in contrast with other materials used in the determination of PCMC [32, 33].

**Table 2:** Comparison of the analytical performance of different sensors in the determination of PCMC

Sensor	Linear range/μM	Sensitivity/μA mM^−1^ cm^−2^	LOD/μM	Reference
4-chloro-3-methylphenol (PCMC)				
GCE	21–210	2.20 × 10^2^	9.3	[35]
GP-CCE	9–29	2.80 × 10^2^	2.71	[36]
CNT-CCE	3–32	1.41 × 10^3^	0.71	[36]
MWCNTs/GCE	14–138	5.91 × 10^3^	8.8	[37]
BDDE	5–100	1.56 × 10^3^	0.34	[32]
UiO-66-NH_2_@PEDOT/GA/GCE	0.6–18	1.87 × 10^0^	0.2	[33]
SNG-C-PANI	2–10	3.05 × 10^3^	1.79	This work
SNG-C/CB-PANI	2–10	5.48 × 10^3^	0.83	This work
4-chlorophenol (4-CP)				
CNTs-OH/PtNPs/RhB/GCE	10–300	8.45 × 10^2^	3.69	[38]
Ag-PTA/CTS/ITO	1–400	2.49 × 10^2^	0.34	[39]
2-CDMA/NiO/NPs/CPE	20–800	2.47 × 10^3^	5.0	[40]
SNG-C/CB-PANI	2–10	2.35 × 10^3^	0.75	This work
2,4-dichlorophenol (2,4-DCP)				
HRP/MWNT/GCE	1–100	7.50 × 10^1^	0.38	[41]
MIP/chitosan/Nafion/GCE	5–100	9.41 × 10^2^	1.6	[42]
Laponite-CPE	0.5–50	2.95 × 10^1^	0.2	[43]
SNG-C/CB-PANI	2–10	1.86 × 10^3^	0.92	This work
2,4,6-trichlorophenol (2,4,6-TCP)				
CNTs-OH/PtNPs/RhB/GCE	5–175	1.85 × 10^3^	1.55	[38]
PPD-CPO-CPE	0.1–10	1.83 × 10^2^	0.1	[44]
MCO@GCN/GCE	0.01–1720	2.25 × 10^1^	0.0068	[45]
SNG-C/CB-PANI	2–10	9.68 × 10^2^	1.06	This work

*GCE* glassy carbon electrode, *GP-CCE* bare carbon ceramic electrode *CNT-CCE* multi-walled carbon nanotubes carbon ceramic electrode, *MWCNTs/GCE* multi-walled carbon nanotubes modified glassy carbon electrode, *BDDE* boron-doped diamond electrode, *UiO-66-NH2@PEDOT/GA/GCE* Zr-based metal-organic framework core-shell structured poly(3,4-ethylenedioxythiophene graphene aerogel modified glassy carbon electrode, *CNTs-OH/PtNPs/RhB/GCE* hydroxylated carbon nanotubes platinum nanoparticles rhodamine B nanocomposite modified glass carbon electrode, *Ag-PTA/CTS/ITO* Ag-phosphotungstic acid composite nanoparticles modified ITO-glass, *2-CDMA/NiO/NPs/CPE* 2-chloro-N0-[1-(2,5-dihydroxyphenyl)methylidene] aniline and NiO nanoparticles based carbon paste electrode, *HRP/MWNT/GCE* horseradish peroxidase multiwalled carbon nanotubes modified glassy carbon electrode, *MIP/chitosan/Nafion/GCE* molecularly imprinted polymer chitosan nafion modified glassy carbon electrode, *PPD-CPO-CPE* poly (ophenylendiamine) chloroperoxidase modified carbon paste electrode, *MCO@GCN/GCE* MnCo_2_O_4_ microcubes embedded on graphitic carbon nitride modified glassy carbon electrode, *LOD* limit of detection

The SNG-C/CB-PANI sensor device has a substantial advantage versus other analogous sensors: no cleaning or later treatment is needed after analyte measurements due to antifouling properties. Sonogel–Carbon electrodes containing carbon black were developed by Pigani et al. and applied in the analysis of caffeic acid in instant coffee samples. However, an electrochemical cleaning process of 6 cycles (50 mV/s) with CV in KOH solution was necessary after each measurement [34]. Similar harsh conditions were used for cleaning step in the analysis of PCMC with the SNG-C-PANI sensor device, with 15 sweeps using CV [3]. These surface renewal procedures extend the analysis time and make it more complex. The absence of this strategy in the application of SNG-C/CB- PANI leads to extremely simple, reproducible, low-cost, and fast analyses.

Additionally, the calibration of other chlorophenols, 4-chlorophenol (4-CP), 2,4-dichlorophenol (2,4-DCP), and 2,4,6-trichlorophenol (2,4,6-TCP), was also carried out with the developed material. The analytical parameters of quality of the sensor are presented in Table S2. RSD values under 5% were obtained for all analytes tested, suggesting the avoiding of surface fouling phenomena, which can be ascribed to the modification of the material with CB previously reported in this work. LODs are of the same level that the one of PCMC, whereas the sequence of sensitivity is PCMC > 4-CP > 2,4-DCP > 2,4,6-TCP. Furthermore, very satisfactory reproducibility and mechanical renewability are displayed by the sensor towards these analytes. In section S4 from Electronic Supplementary Material, full repeatability, reproducibility and mechanical renewal studies can be found.

Also, a brief comparison of the analytical parameters of the modified sensor toward these additional analytes and the performance of other sensors reported in the bibliography are shown in Table 2. The sensitivities displayed by the SNG-C/CB-PANI sensor towards 4-CP and 2,4-DCP are higher or similar than the reported for other sensors. In the case of 2,4,6-TCP, the sensitivity of the modified sensor is lower than the corresponding to another reported sensor. Concerning LOD, similar values were obtained in comparison with the rest of the sensors. In conclusion, a simple modified ceramic material synthesized by fast and cheap methods can be employed as electrochemical transducer of an analytical sensor with great performance due to its excellent antifouling properties in the determination of diverse chlorophenols. However, the similar oxidation potential of the chlorophenols observed in this study reveals the individual analysis of these compounds in complex samples is unattainable in the presence of analogous phenolic species. Hence, in the case of high multiphenolic presence, an interesting approach for the determination of these contaminants could be the estimation of a total phenols index that would provide an approach to the levels of these compounds in studied samples, maybe via chemometric tools. The SNG-C/CB-PANI can be applied for this purpose due to their excellent antifouling properties and electroanalytical performance, although further studies need to be carried out in this sense.

#### Interference study

**Fig. 4:**
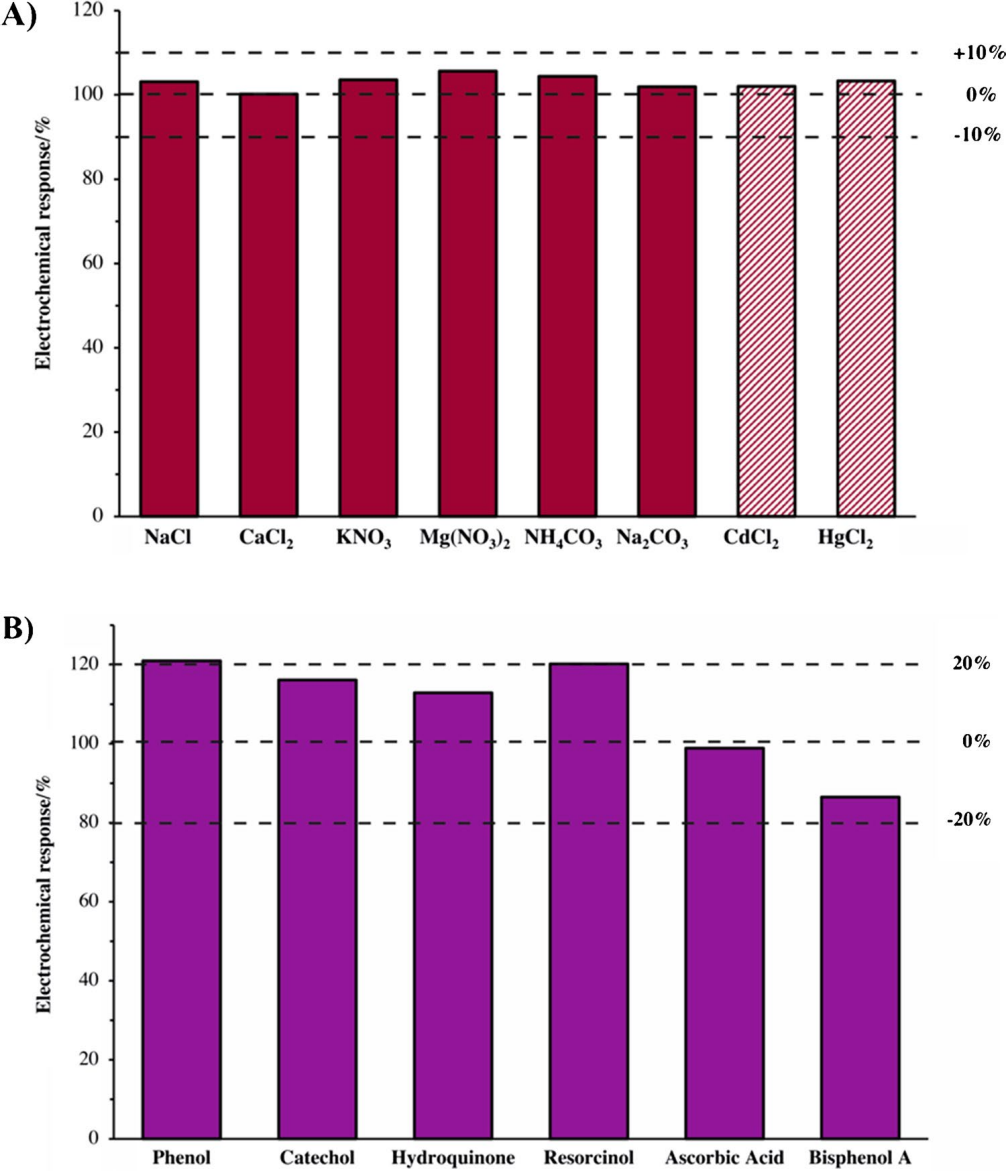
Interference of different compounds in the oxidation signal of 7 μM of PCMC recorded with a SNG-C/CB-PANI electrode in 0.1 M ABS at pH 4 with 0.5 M. **A** Interferences concentration was 700 μM (bold chart) and 70 μM (dashed chart). **B** Interferences concentration was 7 μM

With the aim to assess the electroanalytical performance of the electrochemical sensor developed in this work, the influence of a wide range of ionic and metallic salts and other electroactive (mainly phenolic) species in the determination of PCMC was investigated. Accordingly, the following expression was used (Eq. 1):

1 $$Interference\;\left(\%\right)=\left|\;\left(IPCMC-I\left(PCMC+Int.\right)\right)\;/\;IPCMC\;\right|\times100$$

where I_PCMC_ and I_PCMC+Int_ are the current peaks corresponding to PCMC oxidation in the absence and presence of the interferent evaluated, respectively. The results of this study are shown in Fig. 4. On one hand, notably, none of the tested salts had an interference effect higher than 6% in the signal of PCMC oxidation (Fig. 4A). On the other hand, the other phenols, which most of them can be found in industrial waste alongside chlorophenols, had a positive interference on the PCMC signal, with 10–20% variation (Fig. 4B). Additionally, ascorbic acid had a negligible modification of the PCMC signal, whereas bisphenol A had a negative interference of ca. 14%. These results clearly suggest that most species usually found in real water samples do not seem to interfere dramatically in the determination of PCMC.

### Determination of PCMC in water samples

Lastly, analysis of real samples was carried out to ascertain the selectivity of the developed sensor and its applicability in complex matrices. Notably, the determination of PCMC in water samples is of great interest due to its role as an important environmental hazard in aquatic ecosystems. The US Environmental Protection Agency (USEPA) specify the water quality criterion for PCMC as a concentration level of 500 μg/L (3.5 μM) [31]. Therefore, spiked untreated water samples from different sources were analyzed. With the aim to establish the applicability of the sensor at the same concentration level stipulated by the USEPA, a concentration spiked of 25 μM is proposed for the samples’ analysis. The concentration values of the samples were validated by HPLC as reference method.

The electrochemical analysis of different water samples enriched with PCMC was carried out by the standard addition method, following the method reported in the Experimental section. Table S3 exposes the experimental results achieved in the analysis of five water samples collected from different sources. Average recovery values, defined as the ratio between the PCMC concentration measured using the electrochemical method and the one measured by using HPLC analytical technique, ranging from 97 to 104% were obtained for all samples analyzed in this study. Moreover, the low standard deviation values calculated demonstrate the repeatability provided by the electrochemical method, previously assessed in other studies. Thus, the applicability of the sensor at concentration levels specified by the USEPA was proved and consequently, the developed SNG-C/CB-PANI sensor can be proposed as a useful alternative to other analytical methods widely employed for the determination of PCMC in natural water samples.

## Conclusions

In this work, a new sensor material based on the bulk modification of a ceramic-PANI composite with CB has been synthesized through a green and fast two-step ultrasound-assisted method. By reducing the amount of graphite powder from 600 to 475 mg, an optimized percentage of 5% of the nanomaterial CB has been also incorporated into the silicon oxide network with no volume contraction. The appropriate integration of CB in the bulk material has been proved through structural and electrochemical techniques, achieving the binding of both the nanomaterial and the polymer in the bulk structure.

The electrochemical sensor device developed showed great performance towards PCMC, displaying a sensitivity of 5.48 × 10^3^ ± 3.05 × 10^2^ μA mM^−1^ cm^−2^ greater than the one achieved with the unmodified material and competitive with those found in the literature. Furthermore, anti-fouling phenomena was exhibited by this material as a consequence of the inclusion of CB in its network, increasing its lifetime and versatility. Furthermore, a significant response of the sensor toward other chlorophenols (4-chlorophenol, 2,4-dichlorophenol and 2,4,6-trichlorophenol) has been reported. Finally, it has been proved that this new device is useful in the determination of PCMC in natural untreated water samples, as referenced by HPLC studies, with recoveries values between 97 and 104%. As far as we know, the developed SNG-C/CB-PANI sensor is the first sensor device that is based on a material which combines the CB nanomaterial and the conducting polymer PANI. The synergetic effect of both materials leads to excellent antifouling and electrocatalytic properties that improve the electroanalytical parameters and allow to simplify sample analysis. This novelty heightens the new sensor device versus more complex and demanding (from the operational point of view) conventional sensors.

## Supplementary information

ESM 1 (20.9 MB)
