# Supplementary material for: Incorporation of carbon black into a sonogel matrix: improving antifouling properties of a conducting polymer ceramic nanocomposite
Source: Mikrochim Acta. 2023 Apr 4;190(5):168. doi: 10.1007/s00604-023-05740-z (PMC10070287; doi:10.1007/s00604-023-05740-z)
Supplement: Supplementary file 1 — ESM 1 (20.9 MB) [file 604_2023_5740_MOESM1_ESM.docx]

**Electronic** **Supplementary Material**

*Microchimica Acta*

**Incorporation of Carbon black into a sonogel matrix: improving antifouling properties of a conducting polymer ceramic nano-composite**

Alfonso Sierra-Padilla^1^, David López-Iglesias^1^, Paloma Calatayud-Macías^1^, Juan José García-Guzmán^2^, José María Palacios-Santander^1*^, Laura Cubillana-Aguilera^1^

1 Department of Analytical Chemistry, Institute of Research on Electron Microscopy and Materials (IMEYMAT), Faculty of Sciences, Campus de Excelencia Internacional del Mar (CEIMAR), University of Cadiz, Campus Universitario de Puerto Real, Polígono del Río San Pedro S/N, 11510 Puerto Real, Cadiz, Spain; alfonso.sierra@uca.es (A.S.-P.)(ORCID: 0000-0002-7353-7971); paloma.catalayudmacias@alum.uca.es (P.C.M.); david.lopeziglesias@inibica.es (D.L.-I.)(ORCID: 0000-0001-8278-6585); laura.cubillana@uca.es (L.C.-A.)(ORCID: 0000-0002-3559-2697)

2 Instituto de Investigación e Innovación Biomédica de Cadiz (INiBICA), Hospital Universitario ‘Puerta del Mar’, Universidad de Cadiz, 11009 Cadiz, Spain; juan.garcia@inibica.es (J.J.G.G.)(ORCID: 0000-0002-3124-0141)

* Correspondence: josem.palacios@uca.es (J.M.P.S.)(ORCID: 0000-0001-5407-1208)

**Supplementary Information**  **S2**

**Supplementary Figures**  **S4**

**Supplementary Tables** **S8**

**Supplementary Information**

1. **Study of the effect of pH**

The influence of the pH on the electrooxidation of PCMC was evaluated. **Figure S4A** displays the differential pulse voltammograms recorded with the developed sensor in presence of PCMC in three buffer solutions at different pH values. Interestingly, the oxidation peak potential shifts to more positive potential values when decreasing the pH of the solution, which suggest the participation of protons in the electrochemical oxidation.

The electrochemical response after performing five successive measurements using the three buffer solutions were studied (**Figure S4B**). The oxidation peak current is 5-fold higher at the acid pH, indicating greater electroactivity in this medium. Moreover, a significant decrease of the current peak with each measurement is observed in the solutions with pH 10 and 7, showing an RSD of 20% and 17%, respectively. Contrarily, no clear tendency on the response can be observed at pH 4, with an RSD lower than 2%. In view of these results, ABS at pH 4 was selected as the most suitable electrolytic medium for the determination of PCMC with the developed sensor.

1. **Capacities study**

The electrochemical behaviour of the materials in free-analyte buffer solution were studied to calculate their capacities. CV voltammograms were recorded out in 0.1 M PBS at pH 7 from –0.4 to +0.7 V at different scan rate values in the range of 10-200 mV·s^-1^. Regarding the observed capacity (C_obs_), it can be estimated as the ratio between the average density current and the scan rate, using 100 mV·s^-1^ as the reference scan rate [1]. On the other hand, the double-layer capacity (C_dl_) can be determined from the regression line of the representation of the average density current versus the scan rates values used. Thus, the calculated capacity values for SNG-C-PANI electrodes were 1086 ± 345 µF/cm^2^ and 564 ± 116 µF/ cm^2^, respectively for C_obs_ and C_dl_. In the case of SNG-C/CB-PANI electrodes, the value of C_obs_ was 588 ± 28 µF/ cm^2^, whereas the value of C_dl_ was 310 ± 15 µF/ cm^2^. As it can be observed, SNG-C/CB-PANI material provided lower capacity values. This can be understood as in this material a higher charge is available to oxidize and/or reduce the analytes, so an improvement in the electroanalytical performance may be expected in comparison with the unmodified sensor [2].

1. HPLC methodology

High-performance liquid chromatography (HPLC) was used as reference method for the analysis of PCMC-fortified water real samples. These analyses were carried out in a HPLC system composed of a HITACHI Chromaster 5110 Pump and a MERCK Differential Refractometer RI-71. The column used was a Reverse Phase LiChrosorb® 18, 10 μm particle size, without temperature control. For this validation process, the mobile phase used was methanol-water with 1% formic acid aqueous solution (10:90, v/v) in isocratic elution. A flow rate of 1.0 mL/min was used and the injection volume of samples was 200 μL. Firstly, a calibration plot was obtained using 1, 2, 3 and 4 μM PCMC stock solutions. Then, 25 µM PCMC-fortified samples were diluted ten times to obtain 2.5 µM PCMC solutions. Each diluted samples were injected three separated times to determine the geometric area of the peak and the concentration was calculated with the calibration plot.

1. **Study of repeatability, reproducibility and mechanical renewal**

The repeatability provided with the developed device was studied. The coefficient of variation of the peak heights, considering five successive measurements in 7 µM PCMC solution, was less than a 2%. Due to this excellent value, it can be concluded that fouling phenomena, ascribed to the electrooxidation of phenols, is minimized with the developed device. These results diverge form the observations of a previous study carried out with the unmodified SNG-C-PANI, where the electrochemical renewal constitutes an essential step to achieve acceptable repeatability values [3].

Reproducibility was also investigated using three different electrodes under the same conditions. The variability of the electrochemical responses provided with each one was also tested by triplicate. Importantly, the average electrochemical response provided by the electrodes were remarkably similar, with a coefficient of variation around 2.8%, so the electrochemical behavior of different electrodes of the same material is almost invariant.

Additionally, mechanical renewability of the electrode surface was studied to increase the lifetime of the sensor. Briefly, nine measurements with one electrode were performed, polishing its surface after three of them. Altogether, the coefficient of variation was lower than 1%, suggesting that analytical response after renewal was remarkably similar and that the electrode performance no suffer great changes when its surface was renewed.

**References**

1. Gun G, Tsionsky M, Lev O (1994) Voltammetric studies of composite ceramic carbon working electrodes. Anal Chim Acta 294:261–270. https://doi.org/10.1016/0003-2670(94)80309-9

2. Ajaero C, Abdelrahim MYM, Palacios-Santander JM, et al (2012) Comparative study of the electrocatalytic activity of different types of gold nanoparticles using Sonogel-Carbon material as supporting electrode. Sensors Actuators, B Chem 171–172:1244–1256. https://doi.org/10.1016/j.snb.2012.06.087

3. López-Iglesias D, Fanelli F, Marchi L, et al (2022) Ceramic polyaniline-carbon composite obtained by ultrasound-assisted sol–gel route: Electrochemical performance towards environmental pollutants. J Electroanal Chem 905:115971. https://doi.org/10.1016/j.jelechem.2021.115971

**Supplementary Figures**


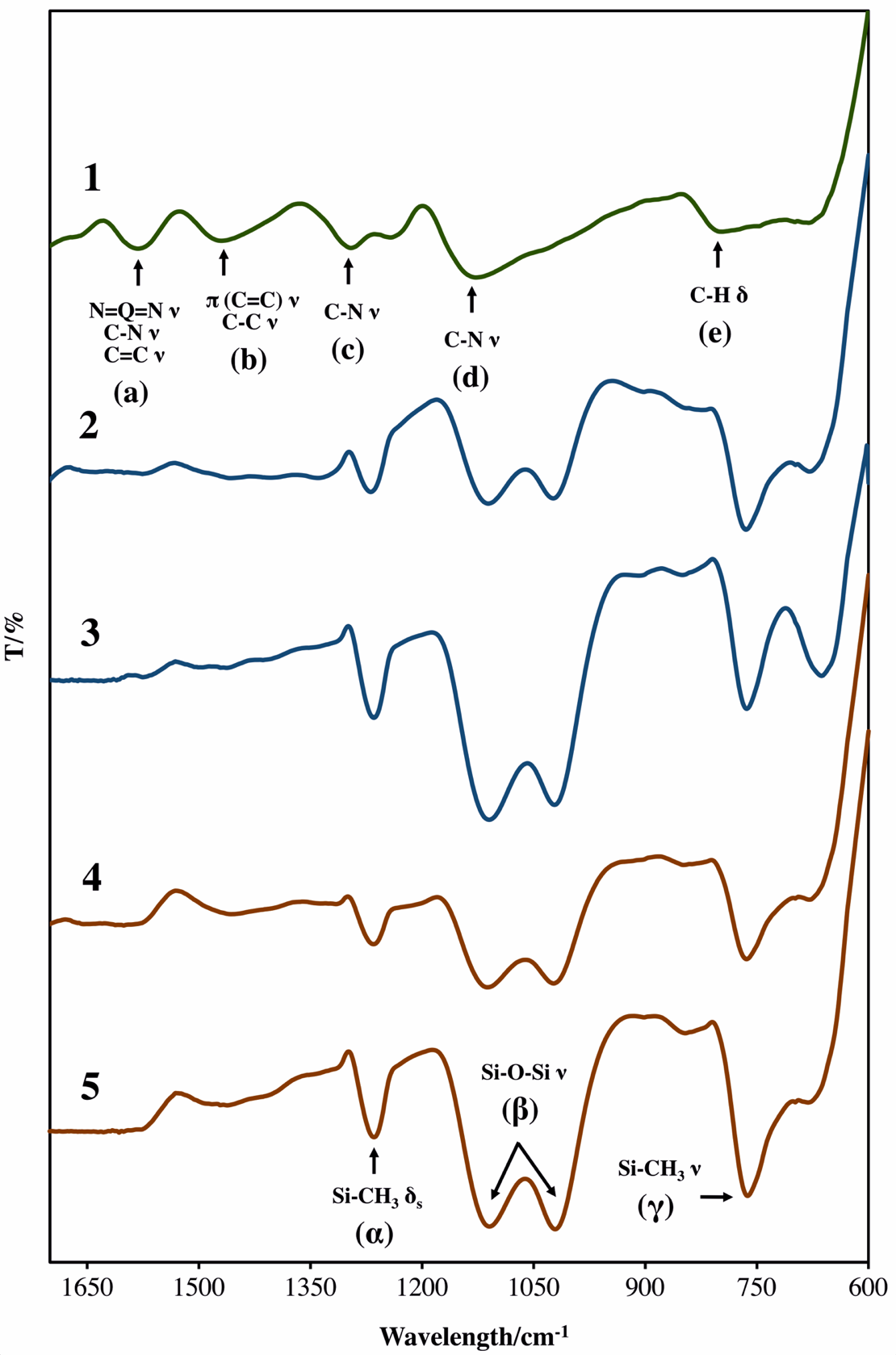


**Fig. S1** FTIR spectra recorded with different samples: (1) PANI; (2) SNGC; (3) SNG-C/CB; (4) SNG-C-PANI; and (5) SNG-C/CB-PANI materials.


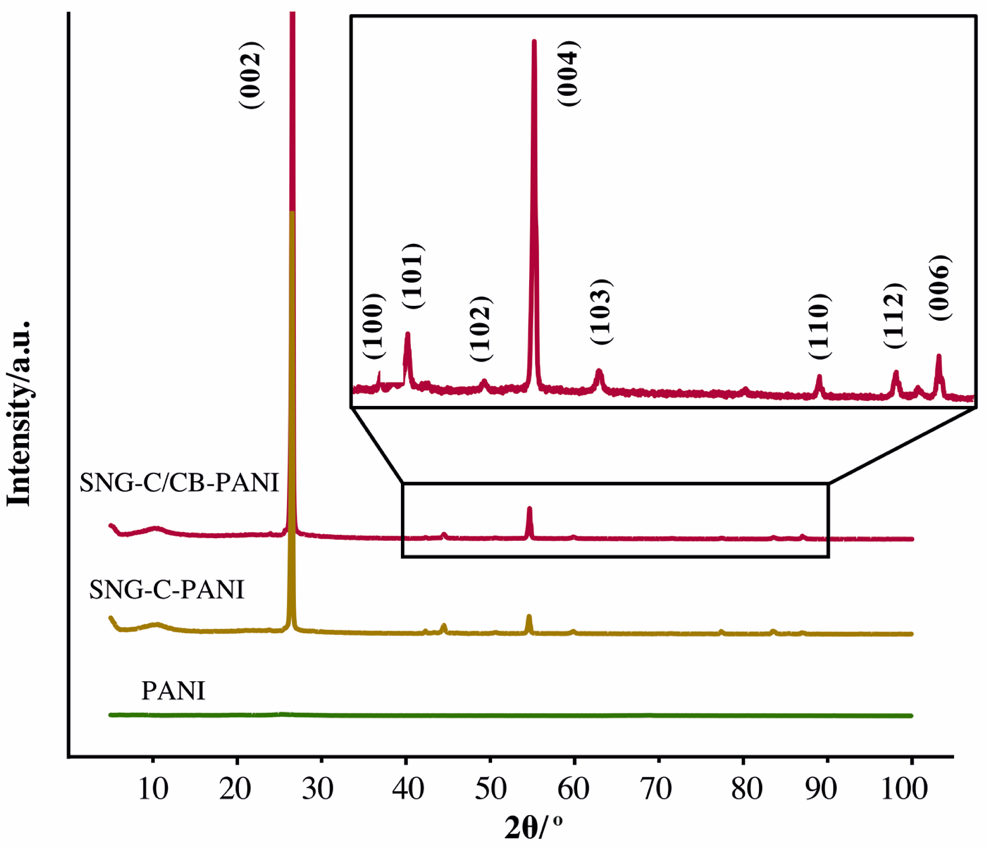


**Fig. S2.** Diffractograms corresponding to PANI, SNG-C-PANI and SNG-C/CB-PANI materials.


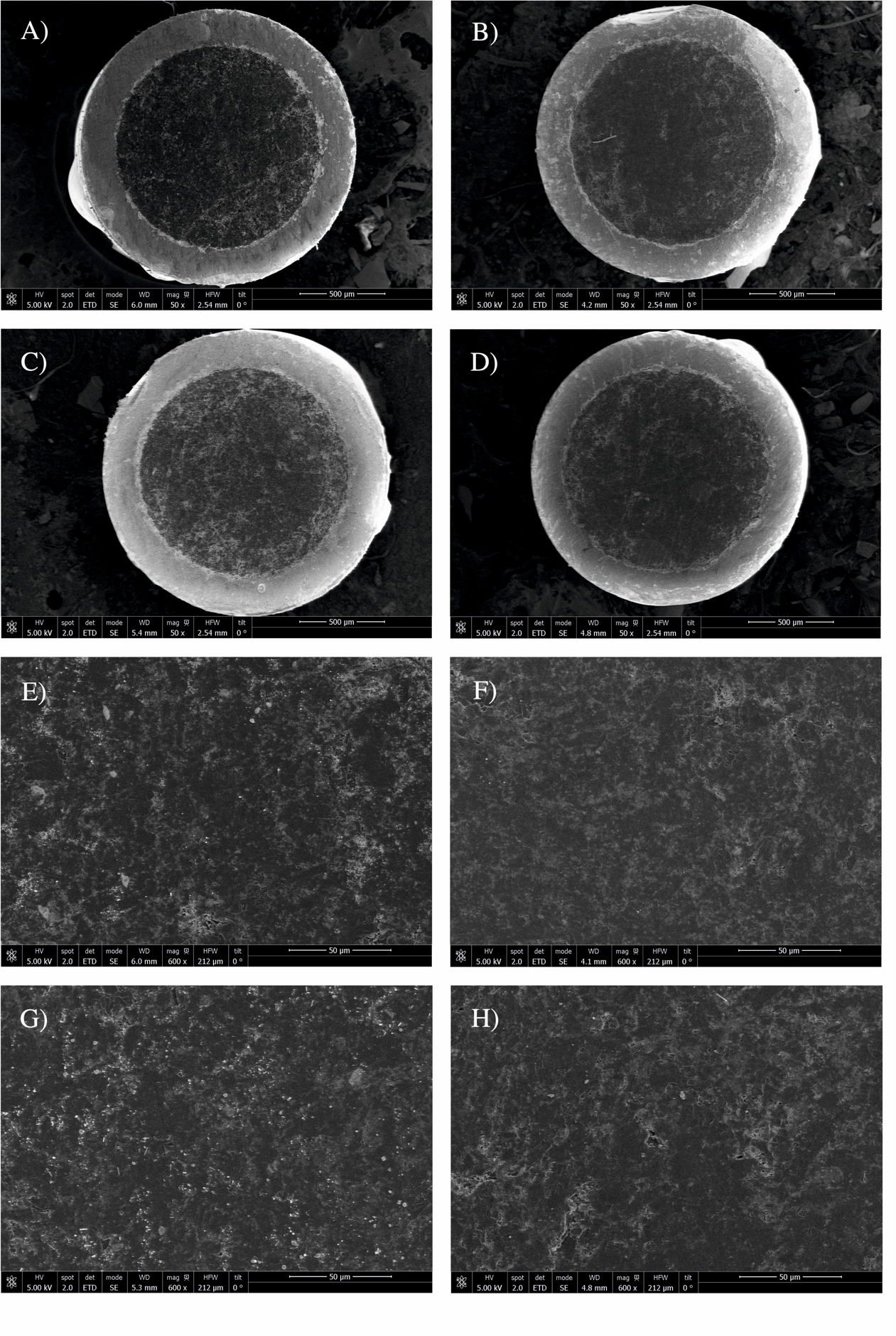


**Fig. S3** SEM micrographs corresponding to SNG-C-PANI electrodes: (A, E) non-used and (B, F) used; and SNG-C/CB-PANI electrodes: (C, G) non-used and (D,H) used. Micrographs (A, B, C, D) were taken with 50× magnification, while (E, F, G, H) were taken with 600× magnification.


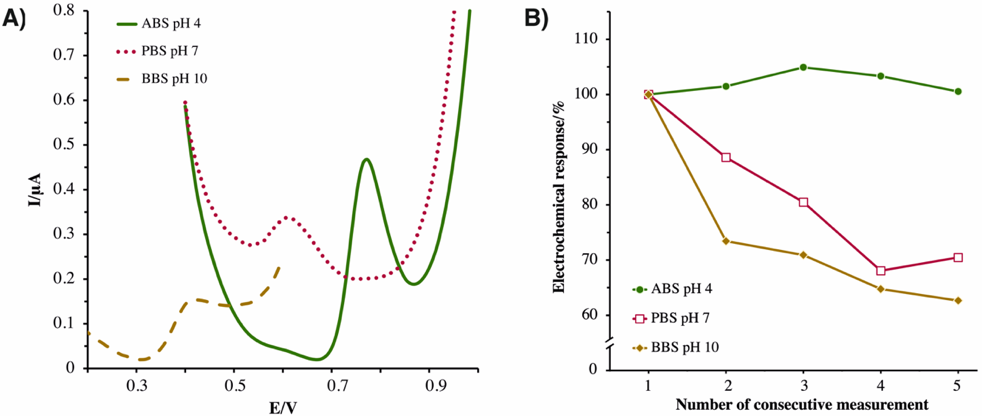


**Fig. S4** (A) Differential pulse voltammograms and (B) electrochemical responses of successive measurements recorded with a SNG-C/CB-PANI electrode in presence of 7 µM PCMC in three different buffer solutions: ABS pH 4 (•), PBS pH 7 (****) and BBS pH 10 (♦). 0.5 M KCl was used as supporting electrolyte in any case.

**Supplementary Tables**

| **Table S1** Electrochemical parameters obtained with a SNG-C/CB-PANI electrode in presence of 5 mM of potassium hexacyanoferrate (II) in 0.5 M of KNO_3_. | | | | | | |
| --- | --- | --- | --- | --- | --- | --- |
| **v (mV/s)** | **I_a_ (µA)** | **I_c_ (µA)** | **I_a_/I_c_** | **E_a_ (V)** | **E_c_ (V)** | **E_a_-E_c_ (V)** |
| 10 | 3.46 | 3.26 | 1.06 | 0.298 | 0.210 | 0.088 |
| 25 | 5.03 | 4.86 | 1.04 | 0.303 | 0.205 | 0.098 |
| 50 | 6.68 | 6.44 | 1.04 | 0.308 | 0.205 | 0.103 |
| 75 | 7.82 | 7.49 | 1.04 | 0.308 | 0.200 | 0.108 |
| 100 | 8.65 | 8.39 | 1.03 | 0.313 | 0.195 | 0.118 |
| 125 | 9.45 | 9.05 | 1.04 | 0.313 | 0.195 | 0.118 |
| 150 | 10.15 | 9.48 | 1.07 | 0.318 | 0.195 | 0.123 |
| 175 | 10.73 | 9.76 | 1.10 | 0.322 | 0.195 | 0.127 |
| 200 | 11.03 | 10.52 | 1.05 | 0.322 | 0.191 | 0.131 |

| **Table S2** Analytical parameters of quality of the SNG-C/CB-PANI sensor device in the determination of diverse chlorophenols. Linear range from 2 to 10 µM in all cases. | | | | | | | |
| --- | --- | --- | --- | --- | --- | --- | --- |
| **Sensor** | **Working potential (V)** | **R^2^** | **Sensitivity (µA · mM^-1^ · cm^-2^)** | **LOD (µM)** | **Repeatability (%)** | **Reproducibility (%)** | **Mechanical renewability (%)** |
| 4-chloro-3-methylphenol | 0.78 | 0.996 | 5459 | 0.83 | 1.24 | 2.79 | 0.20 |
| 4-chlorophenol | 0.80 | 0.996 | 2349 | 0.75 | 1.89 | 4.01 | 2.83 |
| 2,4-dichlorophenol | 0.79 | 0.994 | 1862 | 0.92 | 1.99 | 2.47 | 3.78 |
| 2,4,6-trichlorophenol | 0.80 | 0.993 | 968 | 1.06 | 3.61 | 4.40 | 4.69 |

**Table S3.** Experimental results from the analysis of PCMC in spiked water samples.

| **Sample** | **C_PCMC_ determined by HPLC/µM** | **C_PCMC_ determined by the sensor ± SD^1^/µM** | **Recovery^2^ ± SD^1^/%** |
| --- | --- | --- | --- |
| Tap water | 25.3 | 25.0 ± 0.4 | 98.8 ± 1.7 |
| El Puerto de Santa Maria well water | 25.7 | 26.1 ± 0.3 | 101.8 ± 1.3 |
| Chiclana de la Frontera well water | 26.3 | 26.1 ± 0.4 | 99.4 ± 1.6 |
| Igualeja spring water | 25.0 | 26.4 ± 0.5 | 105.5 ± 1.9 |
| Los Hurones reservoir water | 26.9 | 26.5 ± 1.2 | 98.3 ± 4.4 |

^1^ SD: Standard deviation (n=3)

^2^ Recovery: Error ratio between C_PCMC_ determined by the developed SNG-C/CB-PANI sensor device and C_PCMC_ determined by HPLC.
